# Cryptotanshinone enhances the effect of Arsenic trioxide in treating liver cancer cell by inducing apoptosis through downregulating phosphorylated- STAT3 in vitro and in vivo

**DOI:** 10.1186/s12906-016-1548-4

**Published:** 2017-02-10

**Authors:** Li Shen, Guangshun Zhang, Zhaohuan Lou, Guanhua Xu, Guangji Zhang

**Affiliations:** 1 College of Basic Medical Science, Zhejiang Chinese Medical University, 548 Bin Wen Road, Hangzhou, 310053 Zhejiang Province China; 2 Center for post-doctoral studies, China Academy of Chinese Medicine Science, Beijing, China; 3 College of pharmacy, Zhejiang Chinese Medicine University, Hang Zhou, People’s Republic of China; 4 Institute of Pharmacology, Zhejiang Chinese Medicine University, Hang Zhou, People’s Republic of China; 5 First People’s Hospital of Xiaoshan District in Hangzhou, Hang Zhou, China

**Keywords:** Arsenic trioxide, Cryptotanshinone, Cell apoptosis, Liver cancer

## Abstract

**Background:**

Arsenic trioxide (ATO) is approved for treating terminal-stage liver cancer in China. Cryptotanshinone (CT), a STAT3 inhibitor, has exhibited certain anti-tumor potency; however, the use of CT enhanced ATO for treating liver cancer has not been reported. Here we try to elucidate how CT could enhance the efficacy of ATO for treating liver cancer and its correlation to STAT3 in vitro and in vivo.

**Methods:**

Cell viability of ATO combined with CT was assessed by ^1^MTT assay. Cell apoptosis induced by ATO combined with CT was detected by Annexin V/PI staining and apoptosis-related proteins were detected by western blotting. STAT3-related proteins were analysis by western blotting analysis and Immunofluorescence assays. Efficacy evaluation of ATO combined with CT on xenograft was carried in nude mice and related proteins were analysis by Immunohistochemistry assays.

**Results:**

First we evaluated cell vitality, and our data indicated that the ATO combined with CT showed obvious growth inhibition of Bel-7404 cells compared to ATO or CT alone. Next we found that ATO combined with CT induced cell apoptosis in Bel-7404 cells and upregulated the activation of apoptosis-related proteins cleaved-caspase-3, cleaved-caspase-9, and cleaved-poly(ADP-ribose) polymerase in a time-dependent manner. Next, we found that ATO combined with CT not only inhibited the constitutive levels of phosphorylated-JAK2 and phosphorylated-STAT3^Tyr705^ but did so in a time-dependent manner. We also found that ATO combined with CT reversed the upregulated expression of phosphorylated-STAT3^Tyr705^ stimulated by interleukin-6 and downregulated STAT3 direct target genes and the anti-apoptotic proteins Bcl-2, XIAP, and survivin but obviously upregulated the promoting apoptosis proteins Bak,.In vivo studies showed that ATO combined with CT decreased tumor growth. Tumors from ATO combined with CT–treated mice showed decreased levels of phosphorylated-STAT3^Tyr705^ and the anti-apoptotic protein Bcl-2 but an increased level of pro-apoptotic protein Bax.

**Conclusions:**

Our study provides strong evidence that CT could enhance the efficacy of ATO in treating liver cancer both in vitro and in vivo. Downregulation of phosphorylated-STAT3 expression may play an important role in inducing apoptosis of Bel-7404 cells.

## Background

Primary hepatocellular carcinoma (HCC) is one of the most common malignancies worldwide [1], but only 10–30% of patients are surgical candidates [2]. Chemotherapy is a major terminal-stage liver cancer treatment, but the existing chemotherapy regimens have problems such as a poor curative effect and adverse reactions. Therefore, the identification of new treatment methods to improve survival rates is a critical need.

Arsenic trioxide (ATO), a major component of traditional Chinese medicine, was first used to treat acute promyelocytic leukemia. Some researchers propose that ATO activates the caspase cascade and induces production of reactive oxygen species, resulting in apoptosis [3]. Although low-dose ATO was granted approval by the National Drug Administration in China in 2004 for treating terminal-stage liver cancer, it failed to show a therapeutic effect at an endurable dose in a recent phase II clinical trial [4]. The ATO antitumor effect on liver cancer and glioma solid tumors has been clarified in vivo and in vitro [5, 6]. ATO was shown to lead the mitochondrial permeability transport hole open, cytochrome C release involved in mitochondrial apoptosis pathway. However, the tumor cells of the ERK and AKT pathways as well as nuclear factor-kappaB (NF-ĸβ) and abnormal STAT3 kinase activation, resulting in decreased ATO drug sensitivity [7–9]. Furthermore, some scholars considered using ATO with other drugs to enhance its antitumor effect. The co-treatment of oridonin and As_2_O_3_ induced reactive oxygen species–mediated downregulation of Akt and XIAP and inhibited NF-ĸβ activation in HCC cells (Chen et al, [9]). Genistein synergized with a low dose of ATO (2.5 mg/kg) inhibit the growth of HepG2 tumors, by downregulating Bcl-2 expression, upregulating Bax expression, enhancing the activation of caspase-9 and -3, and increasing the release of cytochrome c [10]. Metformin enhanced both the proliferation-inhibiting and apoptosis-inducing effects of ATO on the HepG2 and Bel7402 HCC cell lines by involving metformin-induced downregulation of Bcl-2 [11].

To enhance the efficacy of ATO in treating liver cancer, we focus on cryptotanshinone (CT), a major tanshinone isolated from *Salvia miltiorrhiza* that has been used for the treatment of coronary artery disease, hyperlipidemia, acute ischemic stroke, and Alzheimer’s disease [12–14]. CT has confirmed ability to inhibit STAT3 phosphorylation [15, 16]. Several groups recently reported that CT could arrest the cell cycle and induce apoptosis in several cancer cell lines [17–19]. CT can inhibit the viability of human SMMC-7721 hepatoma cells, which is related to the reduced expression of MAP2K1 mRNA [20].

Cryptotanshinone has also demonstrated sensitizing effects to a broad range of anti-cancer agents including Fas/Apo-1, tumor necrosis factor-α, cisplatin, etoposide, and 5-FU by inducing ER stress, highlighting its therapeutic potential in the treatment of human hepatoma and breast cancer (Park et al. [19]).

Aberrant activation of JAK/STAT3 signaling has been found in many tumors [21–23]. In particular, STAT3 participates in the initiation, development, and progression of human cancers by inducing STAT3 downstream genes that encode anti-apoptotic proteins, cell cycle regulators, and angiogenic factors such as Bcl-xl and cyclin D1 [24, 25]. Cytokines of the interleukin-6 (IL-6) family, including IL-6, are potent activators of the JAK/STAT3 pathway and predominantly activate STAT3 via JAK1 and JAK2. IL-6 caused STAT3 kinase activation, resulting in anti-apoptotic Bcl-2 expression and inhibiting of apoptosis proteins such as Bcl-xl and Mcl-1. The inhibition of constitutive STAT3 activation in malignant cells can suppress Bcl-xl and Mcl-1 genes [26].

According to the above results, we hypothesized that CT could enhance the efficacy of ATO for treating liver cancer and that phosphorylated-STAT3 may play a key role. Here we try to elucidate how CT could enhance the efficacy of ATO for treating liver cancer and its correlation to STAT3 in vitro and in vivo. Our research aimed to provide terminal-stage liver cancer patients with more effective treatment.

## Method

### Cell lines

The Bel-7404 gastric cancer cell line was obtained from the Center Laboratory of Zhejiang Provincial Hospital of TCM, China, and cultured in RPMI-1640 supplemented with 10% fetal bovine serum.

### Reagents

Hematoxylin and 3-(4,5-dimethyl-2-thiazolyl)-2,5-diphenyl-2-H-tetrazolium bromide (MTT) were purchased from Sigma. Arsenic trioxide for injection was purchased from Double Heron Pharmaceutical Co., LTD. Cryptotanshinone was purchased from Chengdu Must, Bio-technology Co., LTD. Antibodies against cleaved-caspase-3, cleaved-caspase-9, cleaved poly(ADP-ribose) polymerase, Bax, Bak, XIAP, Mcl-1, Bcl-2, Bcl-xl, survivin, phosphorylated-JAK2, and phosphorylated-STAT3^Tyr705^ were purchased from Cell Signaling Technology, while β-actin antibody was purchased from Sigma-Aldrich. An Annexin V/PI binding kit was purchased from Santa Cruz Biotechnology, Inc. RIPA Lysis Buffer and a BCA Protein Assay Kit were purchased from Beyotime. Immobilon ECL was purchased from Millipore. Rhodamin-labeled goat anti-mouse immunoglobulin G (IgG) and DAPI were obtained from Hangzhou Dawei Biotech Co., LTD.

### Cell viability analysis

The cells were plated in 96-well plates (3000–4000 cells/well) in triplicate, incubated overnight, and treated with different concentrations of CT (10 μM, 20 μM), ATO (1 μM, 2 μM), or CT (10 μM, 20 μM) combined with ATO (1 μM, 2 μM) for 24 h, and then cell viability was assessed by MTT assay. Briefly, 20 μL of MTT 5 mg/mL was added to each cell plate and the cells were incubated for 4 h. The medium was then removed and 150 μL of dimethylsulfoxide was added. The absorbance was then detected at 490 nm using an enzyme standard instrument (BioTek). Cell viability was normalized to that of untreated cells.

### Annexin V/PI staining to detect apoptosis

Bel-7404 were divided into four groups (5 × 10^5^ cells each): control, CT, ATO, and CT combined with ATO. After treatment for 24 h, apoptosis was detected using the Annexin V/PI binding kit and then analyzed by a Fluorescence Activated Cell Sorter(FACS Calibur; BD Biosciences). The experiments were performed in triplicate.

### Western blotting analysis

Total protein was isolated using RIPA Lysis Buffer, and the concentrations were measured using the BCA Protein Assay Kit. Proteins (30 μg) were separated by sodium dodecyl sulfate–polyacrylamide gel electrophoresis with 10% separation gels and then transferred to polyvinylidene fluoride membranes. The membranes were blocked by 5% non-fat milk in Tris-buffered saline with Tween-20 (0.01% Tween) for 1 h, incubated with primary antibodies overnight at 4 °C, followed by a 1-h incubation with horseradish peroxidase–conjugated secondary antibodies and then developed with Immobilon enhanced chemiluminescence (ECL) [27].

### Immunofluorescence assays

Cells were treated for 24 h and immunofluorescence staining was performed as previously described [28]. We used the primary antibodies and rhodamine-labeled goat anti-mouse IgG as mentioned above. The nuclei were stained with 4,6-diamino-2-phenyl indole(DAPI) 1 mg/mL for 30 min. Fluorescent images were acquired with a laser scanning confocal microscope (Leica).

### Animals

Forty 5-week-old-male BALB/c nude (weight, 20 ± 2 g) were obtained from Shanghai Super B&K Laboratory Animal Corp. Ltd. and raised in the Zhejiang Chinese Medical University Animal Experiment Research Center. Five mice were housed per standard cage in a room maintained at constant temperature and humidity with a 12-h light:dark cycle. The mice were fed a regular sterilized chow diet with water. A laboratory animal management and welfare ethical review was performed by the Laboratory Animal Management and Ethics Committee of Zhejiang Chinese Medical University (ZSLL-2013-019).

### ATO combined with CT treatment of xenograft tumors

Bel-7404 cells were harvested from subconfluent cultures, washed once in serum-free medium, and resuspended in phosphate-buffered saline (PBS). Bel-7404 cells (5.0 × 10^6^) in 200 μL of PBS were injected into the axillary back area under the skin of BALB/C nude mice. When the tumors reached approximately 100 mm^3^, the nude were randomly divided into four groups: control (normal saline, once daily, intraperitoneal [ip] injection), ATO (2.5 mg/kg, once daily, ip injection), CT (100 mg/kg, once daily, ip injection), and ATO (2.5 mg/kg, once daily, ip injection) combined with CT (100 mg/kg, once daily, ip injection). The tumors were measured twice weekly by a vernier caliper and the tumor volume computation formula was V = a × b^2^ × π/6, where V indicated approximate tumor volume, a indicated the tumor’s long diameter, and b indicated the tumor’s short diameter. The mice were euthanized on the second day after 19 days of treatment. Each tumor excised from the mice was weighted and divided into two parts. One part of the tumor tissue was formalin-fixed, while another part was snap-frozen in liquid nitrogen and stored at −80 °C. The tumor inhibition rate (%) was calculated as (1 - average tumor weight of treatment group/the average tumor weight of control group) × 100%.

### Immunohistochemistry assays

In brief, the tissue sections were deparaffinized in xylene, rehydrated with ethanol, hot-repaired by high pressure, and blocked by 30% H_2_O_2_. Next, the tissue sections were incubated with primary antibody (phosphorylated-STAT3^Tyr705^, Bcl-2, and Bax antibodies) overnight at 4 °C (the PBS was replaced with primary antibody in the negative immunohistochemistry control), and then stained with secondary antibody for 1 h at room temperature. The peroxidase reaction was developed with diaminobenzidine and the slides were counterstained with hematoxylin.

### Statistical analysis

Data are presented as mean ± standard deviation (SD) derived from at least three independent experiments. The data were analyzed by one-way analysis of variance followed by the post hoc least significant differences test. *P*-values < 0.05 were considered statistically significant.

## Results

### ATO combined with CT inhibited Bel-7404 cell growth

**Fig. 1:**
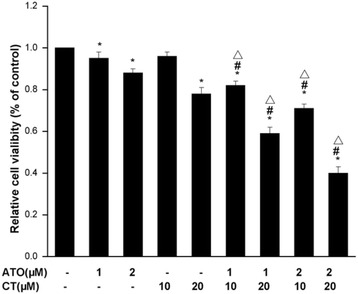
Cell viability analysis. The cell vitality testing of (ATO combined with CT on Bel-7404 cells. The cells were divided into control, CT (10 μM, 20 μM), ATO (1 μM, 2 μM), and CT (10 μM, 20 μM) combined with ATO (1 μM, 2 μM) groups and treated for 24 h and their vitality was determined by 3-(4,5-dimethyl-2-thiazolyl)-2,5-diphenyl-2-H-tetrazolium bromide assays. Each experiment was done in triplicate and the results were averaged. The data represent mean ± SD of triplicate samples from a representative experiment of three independent experiments. *P < 0.05 vs. control; #*P* < 0.05 ATO + CT vs. ATO; Δ*P* < 0.05 ATO + CT vs. CT

Bel-7404 cells were treated with ATO (1 μM, 2 μM), CT (10 μM, 20 μM), or ATO combined with CT for 24 h, and then cell viability was assessed by MTT assay. Cell viability was normalized to control cells. ATO combined with CT treatment had an obvious dose-dependent growth inhibition effect on Bel-7404 cells compared to ATO or CT treatment alone (Fig. 1).

### ATO combined with CT induced liver cancer cell apoptosis

**Fig. 2:**
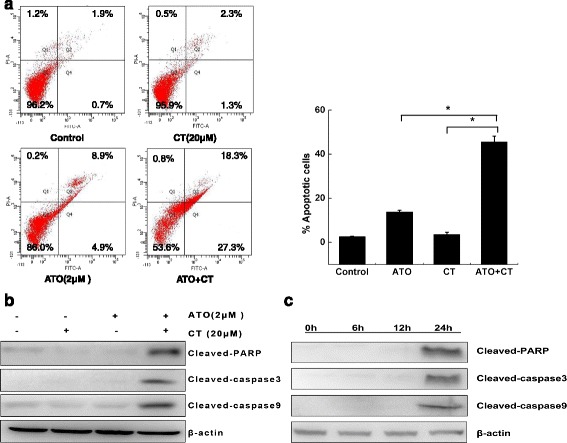
ATO combined with CT induced liver cancer cell apoptosis. **a** Bel-7404 cells were divided into four groups (control, CT, ATO, CT combined with ATO). After 24-h treatment, the cells were stained using an Annexin V/PI binding kit and then analyzed by a FACS flow cytometer. **b** Cells were treated for 24 h, and whole-cell extracts were prepared and analyzed by western blot using antibodies against cleaved-caspase-3, cleaved-caspase-9, and cleaved-PARP. **c** The cells were treated with ATO combined with CT for the indicated times and the expressions of cleaved-caspase-3, cleaved-caspase-9, and cleaved-PARP were analyzed by western blotting. The experiments were performed in triplicate

Annexin V/PI staining was used to investigate whether ATO combined with CT can induce cell apoptosis in Bel-7404 cells. We found that apoptosis was induced by 13.8% ATO (2 μM), 3.6% CT (20 μM), and 45.6% ATO combined with CT (Fig. 2a). Next, we use the western blot method to detect the apoptosis-related proteins cleaved-caspase-3, cleaved-caspase-9, and cleaved-PARP. We found that, although ATO or CT alone had little effect on the activation of cleaved-caspase-3, cleaved-caspase-9, and cleaved-PARP, that of the combined treatment group was highly increased (Fig. 2b). To explore whether this increase was time-dependent, the cells were treated with ATO combined with CT for the indicated time. We found that ATO combined with CT increased the expression of all three proteins in a time-dependent manner (Fig. 2c).

### ATO combined with CT inhibited both endogenous constitutive and Il-6-induced STAT3 activation in Bel -7404 cells

**Fig. 3:**
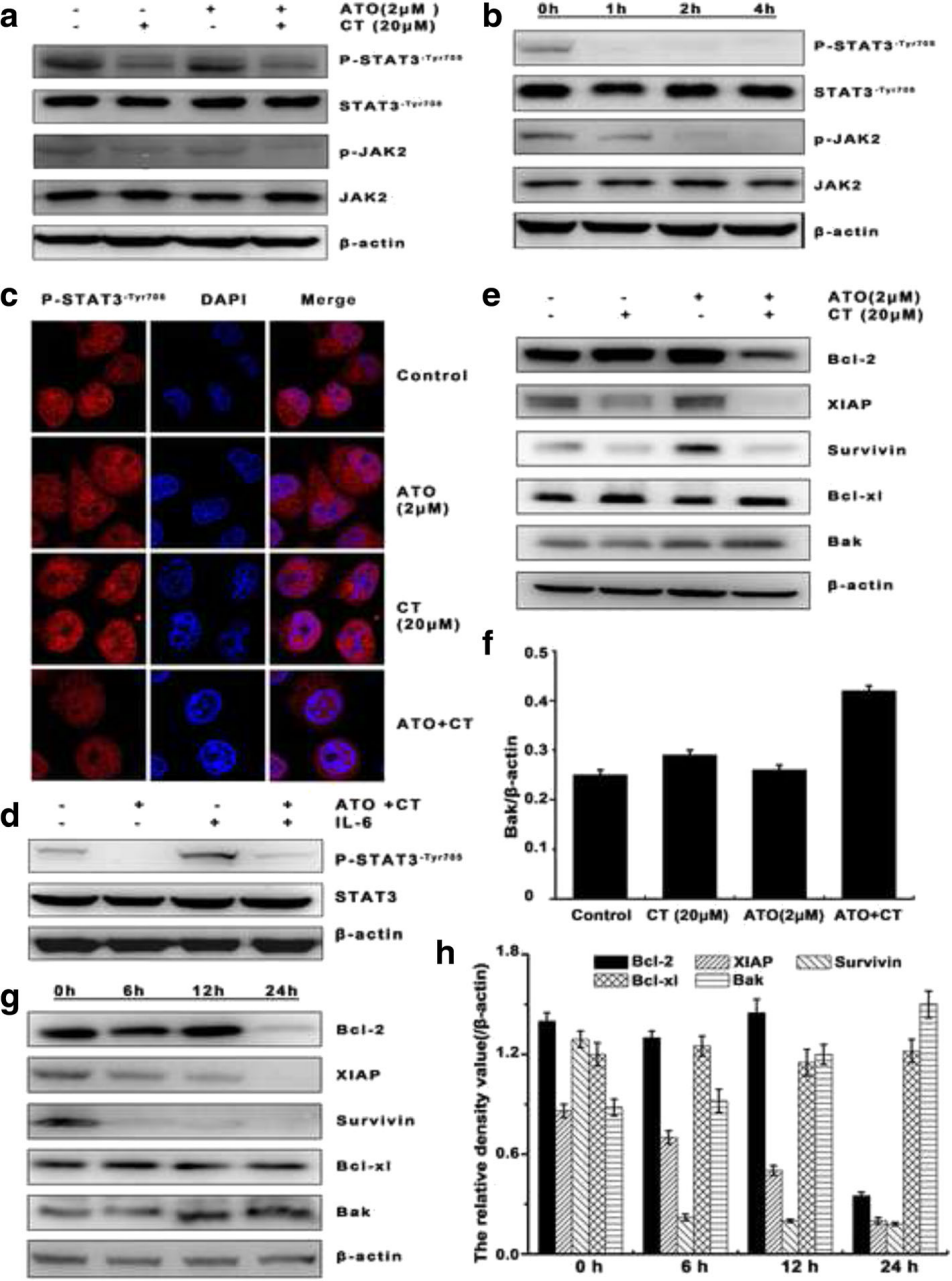
ATO combined with CT inhibited both endogenous constitutive and Il-6–induced STAT3 activation in Bel-7404 cells. **a** Bel-7404 cells were divided into four groups (control, CT, ATO, and CT combined with ATO) and treated for 24 h, while whole-cell extracts were prepared and analyzed by western blotting using antibodies against phosphorylated-JAK2 and phosphorylated-STAT3^-Tyr705^. **b** Cells were treated with ATO combined with CT for the indicated times and the expressions of phosphorylated-JAK2 and phosphorylated-STAT3^-Tyr705^ were detected by western blotting. **c** Laser scanning confocal microscopy was used to investigate the effect of ATO combined with CT on phosphorylated-STAT3-Tyr705 expression a 4 h. The nuclei were stained with DAPI (*blue*), while phosphorylated-STAT3^-Tyr705^ was stained with anti-phosphorylated-STAT3^-Tyr705^ antibody followed by rhodamine-conjugated antibody (*red*). **d** ATO combined with CT reversed the interleukin-6–induced phosphorylated-STAT3^-Tyr705^ upregulation (×800). **e**, **g** STAT3 direct target genes such as anti-apoptotic protein and promoting apoptosis proteins were detected by western blotting and the time-dependent relationships were detected. **f**, **h** Desnisity were measured by quantity one. The bar graphs were drawn based on the relative density

Aberrant activation of JAK/STAT3 signaling has been found in many tumors (Zhang et al. [21]-Hu et al. [23]). To examine whether ATO combined with CT–induced liver cancer cell apoptosis was related to the JAK/STAT3 signaling pathway, we detected the expressions of phosphorylated-JAK2 and phosphorylated-STAT3^-Tyr705^. We found that the combination group not only inhibited the constitutive levels of phosphorylated-JAK2 and phosphorylated-STAT3^-Tyr705^ but did so in a time-dependent manner (Figs. 3a, b). Furthermore, we employed laser scanning confocal microscopy method as a qualitative assay to investigate the effect of ATO combined with CT on phosphorylated-STAT3^-Tyr705^ expression. The nuclei were stained with DAPI (blue), while the phosphorylated-STAT3^-Tyr705^ was stained with anti-phosphorylated- STAT3^-Tyr705^ antibody followed by rhodamine-conjugated antibody (red). We found that the expression of phosphorylated-STAT3^-Tyr705^ influenced by ATO combined with CT was also decreased (Fig. 3c). Studies have shown that cytokines of the IL-6 family, including IL-6, was potent activators of JAK/STAT3 pathway, predominantly activating STAT3 through JAK1 and JAK2 [29]. we evaluated whether ATO combined with CT would affect this signal transduction pathway. As shown in Fig. 3d, phosphorylated-STAT3^-Tyr705^ was upregulated when stimulated by IL-6 10 ng/mL (R&D Systems Inc.) for 4 h. However, this effect was reversed by ATO combined with CT treatment.

To determine whether STAT3 phosphorylation inhibition affected STAT3 target gene expression, we analyzed the expression of selected STAT3 direct target genes. Anti-apoptotic proteins such as Bcl-2, XIAP, survivin, Bcl-xl and the promoting apoptosis proteins Bak was detected by immunoblots. We found that, compared to the control group, the combination group significantly downregulated the expression of anti-apoptotic proteins Bcl-2, XIAP, and survivin but obviously upregulated the pro-apoptosis proteins Bak, whereas Bcl-xl expression had no obvious changes (Fig. 3e, f). We also found that ATO combined with CT regulated apoptosis- related proteins in a time-dependent manner (Fig. 3g, h). All of these results indicate that phosphorylated-STAT3 played a key role in ATO combined with CT–induced liver cancer cell apoptosis.

### ATO combined with CT inhibited xenograft tumor growth on nude mice

**Fig. 4:**
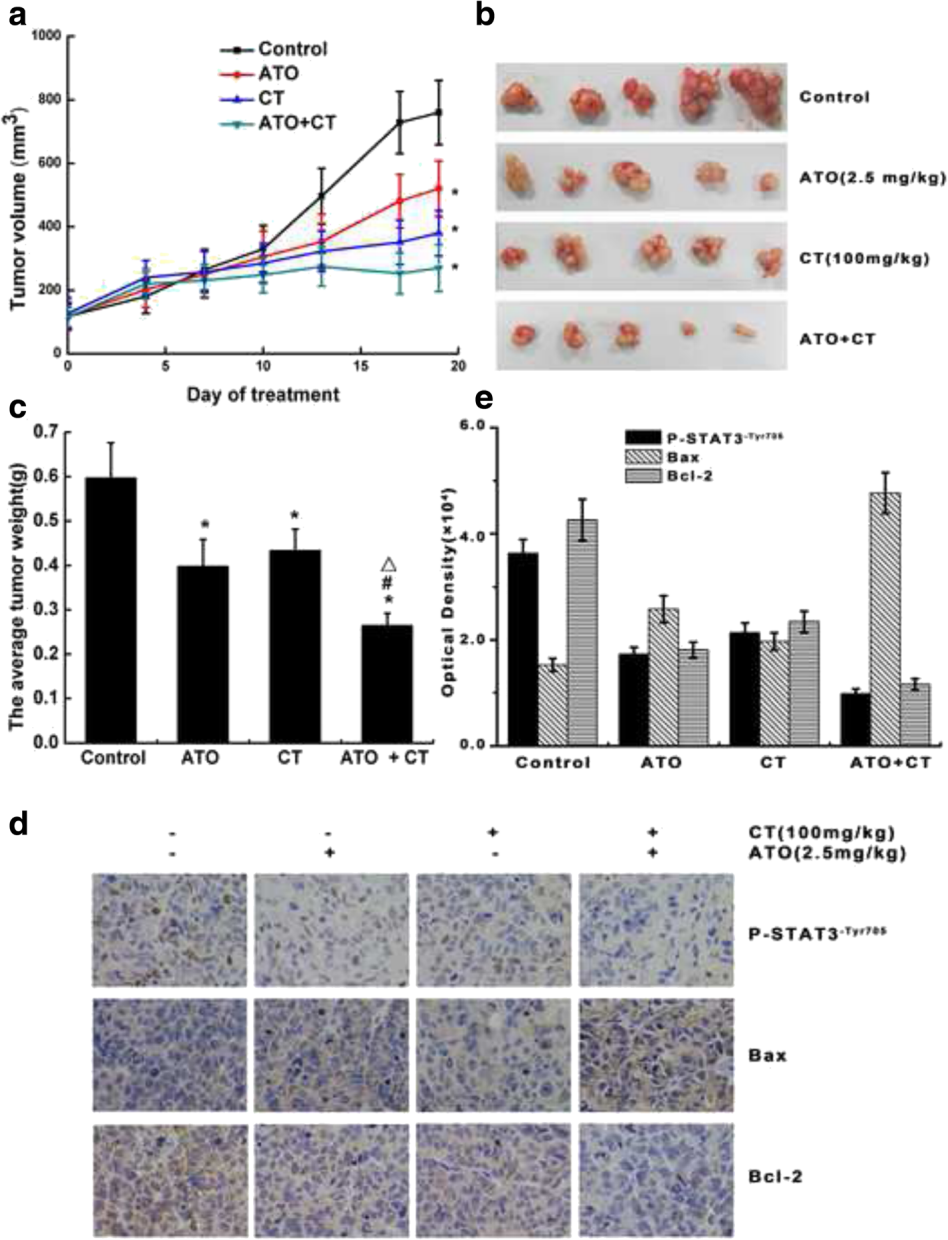
ATO combined with CT inhibited xenograft tumor growth on nude mice. **a** The 4–6-week-old male nude mice were injected subcutaneously with 5 × 10^6^ cells. When the subcutaneous tumors reached approximately 100 mm^3^, the mice were randomly assigned to four groups: control, ATO, CT, and CT combined with ATO. Tumors were measured twice a week by a vernier caliper and the tumor volume growth curve is shown (*P < 0.01 vs. control). **b** The mice were euthanized on the second day after 19 days of treatment. The excised tumors were weighed. Five mice were randomly selected from each group and photographed using a digital camera. **c** The tumor weight was calculated (*P < 0.01 vs. control; #*P* < 0.01 ATO + CT vs. ATO; △*P* < 0.01 ATO + CT vs. CT). **d** The expressions of Phosphorylated- STAT3^-Tyr705^ and the related apoptotic proteins Bcl-2 and Bax were investigated by immunohistochemistry. *Brown*, positive; *blue*, nuclei (×400). **e** Immunohistochemistry results have been quantified by statistical software Image-Pro Plus6.0. Quantifiable results were indicated by mean optical density value

To determine the effect of ATO combined with CT on tumor growth in vivo, we employed the subcutaneous Bel-7404 tumor model. The tumors were randomly assigned into four groups. Tumor volumes were measured every 3–4 days and the excised tumors were weighed at the end of the day. Overall, the results showed a gradual increase in tumor volume in every group. However, compared to the control group, the volumes in the remaining groups tended to increase slowly. On days 14, 17, and 19, the differences between the control group and the other groups were significant. Meanwhile, the combination group presented the slowest growth rate (Fig. 4a). The excised tumors were weighed. Five mice were randomly selected from each group and photographed using a digital camera (Fig. 4b). The tumor weights in the treatment groups were significantly smaller than those of the control group (*P* < 0.01), while those of the combination group were significantly smaller than those of the ATO (*P* < 0.01) and CT (*P* < 0.01) groups (Fig. 4c). We next investigated the expression of phosphorylated-STAT3^-Tyr705^ and the related apoptotic protein Bcl-2 and Bax by immunohistochemistry. Our analyses revealed that tumors from the ATO combined with CT group had decreased levels of phosphorylated-STAT3^-Tyr705^ as well as of the anti- apoptotic protein Bcl-2 compared to the control group. However, the levels of pro-apoptotic protein Bax were significantly increased in the ATO combined with CT group (Fig. 4d, e).

## Discussion

HCC is the most common liver malignancy and a major health problem globally. Targeted therapy of the signal transduction pathway in human malignancies is a recent approach that has shown great promise when used alone or combined with conventional therapies [30]. Here we clarified that CT could enhance the efficacy of ATO in treating liver cancer and that phosphorylated-STAT3 may play a key role.

The use of ATO has been considered with other drugs to enhance its antitumor effect [9–11, 31]. To see whether ATO combined with CT inhibited Bel-7404 cell growth, we first evaluated cell vitality when Bel-7404 cells were treated with different concentrations of ATO or CT. Our data indicated that ATO combined with CT treatment showed obvious growth inhibition compared to the use of ATO or CT alone in a dose-dependent manner.

We then found that ATO combined with CT could induce cell apoptosis rates in Bel-7404 cells and upregulate the activation of apoptosis-related proteins cleaved- caspase-3, cleaved-caspase-9, and cleaved-PARP in a time-dependent manner. These results supported our initial hypothesis that CT could enhance the efficacy of ATO for treating liver cancer. These results were consistent with those of the above reports [9, 10].

As a STAT3 inhibitor, CT has confirmed ability to inhibit STAT3 phosphorylation activity [15, 16]. STAT3 participates in the initiation, development, and progression of human cancers via inducing downstream genes that encode anti-apoptotic proteins, cell cycle regulators, and angiogenic factors such as Bcl-xl and cyclin D1 [17–19]. Next we found that ATO combined with CT not only inhibited the constitutive levels of phosphorylated-JAK2 and phosphorylated- STAT3^-Tyr705^ but did so in a time-dependent manner. We also found that ATO combined with CT reversed the upregulated expression of phosphorylated-STAT3^-Tyr705^ stimulated by IL-6 and downregulated STAT3 direct target anti-apoptotic proteins Bcl-2, XIAP, and survivin while obviously upregulating the pro-apoptotic proteins Bak in Bcl-7404 cells. These results suggest that phosphorylated-STAT3 plays an important role in the ATO enhanced CT–induced liver cancer cell apoptosis in vitro and that downregulating phosphorylated-STAT3 may be key.

Finally, we employed the subcutaneous Bel-7404 tumor model. Our in vivo studies showed that CT enhanced the effect of ATO in reducing tumor growth. Tumors from ATO combined with CT-treated mice showed decreased levels of phosphorylated- STAT3^-Tyr705^ and the anti-apoptotic protein Bcl-2, while levels of the pro-apoptotic protein Bax was increased.

In summary, here we confirmed that CT could enhance the efficacy of ATO in treating liver cancer both in vitro and in vivo. Our results suggest that the downregulation of phosphorylated-STAT3 expression may play an important role in inducing apoptosis of Bel-7404 cells. These results provide a new way of thinking about the use of CT and ATO for treating liver cancer. However, whether CT could reduce the side effects caused by high-dose ATO in treating liver cancer remains to be further elucidated.

## Conclusions

Our study provides strong evidence of the anti-tumor growth potency of ATO combined with CT and that phosphorylated-STAT3 played a key role in ATO combined with CT–induced liver cancer cell apoptosis.

## Abbreviations

Annexin V/PI: Annexin V/propidium iodide
ATO: Arsenic trioxide
Bak: Bcl-2 homologous antagonist/killer
Bax: Bcl-2 Associated X Protein
Bcl-2: B cell lymphoma/lewkmia-2
Bcl-xl: B-cell lymphoma-extra large
CT: Cryptotanshinone
DAPI: 4,6-diamino-2-phenyl indole
ECL: Enhanced chemiluminescence
ERK: Extracellular regulated protein kinase
FACS: Fluorescence activated cell Sorter
HCC: Primary hepatocellular carcinoma
IL-6: Interleukin-6
ip: Intraperitoneal
JAK: Janus kinase
Mcl-1: Myeloid cell leukemia-1
MTT: 3-(4,5-dimethy l-2-thiazolyl) -2,5-diphenyl-2-H-tetrazolium bromide
NF-ĸβ: Nuclear factor-kappaB
PBS: Phosphate-buffered saline
RPMI-1640: Roswell park memorial institute-1640
STAT3: Signal transducer and activator of transcription 3
TCM: Traditional Chinese medicine
V: Volume
XIAP: X-linked inhibitor of apoptosis protein
